# *Lycium barbarum* glycopeptide reduces brain damage following ischemic stroke by inhibiting ferroptosis and oxidation

**DOI:** 10.4103/NRR.NRR-D-24-00747

**Published:** 2025-01-29

**Authors:** Wei Zhang, Moushen Tang, Yujie Wang, Yongxia Huang, Zhexiong Yu, Lihui Zhu, Jian Wang, Kwok-Fai So, Yiwen Ruan

**Affiliations:** 1 Key Laboratory of CNS Regeneration (Ministry of Education), Guangdong-Hong Kong-Macau Institute of CNS Regeneration, Jinan University, Guangzhou, Guangdong Province, China; 2 Ningxia Zhongning Wolfberry (Tianren) Academician Workstation, Yinchuan, Ningxia Hui Autonomous Region, China; 3 Department of Neurology, Guangzhou Xinshi Hospital, Guangzhou, Guangdong Province China; 4 Department of Human Anatomy, School of Basic Medical Sciences, Zhengzhou University, Zhengzhou, Henan Province, China

**Keywords:** antioxidation, anxiety, cerebral ischemic, depression, inflammation, iron overload, *Lycium barbarum* glycopeptide, magnetic resonance imaging, memory, neurological function, neuroprotection

## Abstract

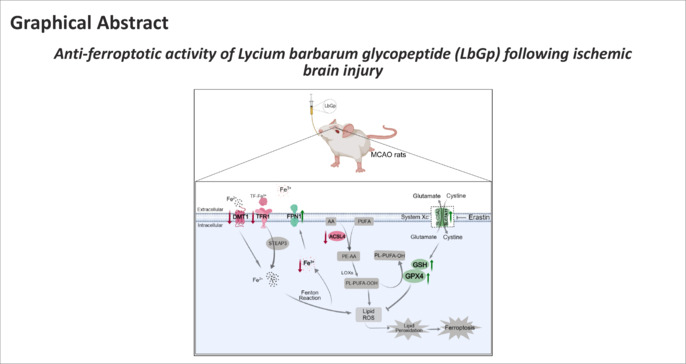

## Graphical abstract

Recent studies have indicated that stroke can lead to neuronal iron overload and lipid peroxidation. *Lycium barbarum* glycopeptide, which has a low molecular weight and potent antioxidant properties, may mitigate ferroptosis in stroke. We hypothesized that *Lycium barbarum* glycopeptide can effectively mitigate iron overload within ischemic neurons due to its robust antioxidant properties. The aims of this study were to investigate the effects of *Lycium barbarum* glycopeptide on ferroptotic damage following brain ischemia and explore the underlying mechanisms. A rat model of middle cerebral artery occlusion was established using the intraluminal filament method, and the rats were treated with *Lycium barbarum* glycopeptide for 7 consecutive days, beginning 24 hours after ischemia. Liproxstatin-1, a ferroptosis inhibitor, and Erastin, a ferroptosis activator, were used as controls. We found that treatment with *Lycium barbarum* glycopeptide resulted in significant reductions in infarct volume (as detected by triphenyltetrazolium chloride staining staining and magnetic resonance imaging) and neuronal death (as measured by Nissl staining), as well as improvements in sensory and motor functions in rats subjected to middle cerebral artery occlusion. Furthermore, treatment with *Lycium barbarum* glycopeptide alleviated anxiety and depression-like behaviors and improved memory. Additionally, *Lycium barbarum* glycopeptide effectively reduced the iron ion content in the ischemic penumbra of the cortex. Moreover, treatment with *Lycium barbarum* glycopeptide downregulated the expression of ferroptotic and oxidant proteins such as transferrin receptor 1, divalent metal transporter 1, and Acyl-CoA synthetase long-chain family member 4 and upregulated the expression of anti-ferroptotic and antioxidant proteins such as ferroportin 1, solute carrier family 7 member 11, glutathione, and glutathione peroxidase 4. However, these beneficial effects were reversed when ferroptosis was induced with the activator Erastin. Therefore, the positive effects of *Lycium barbarum* glycopeptide in ischemic stroke are likely mediated through activation of the anti-ferroptotic pathway and the antioxidative System Xc-glutathione-glutathione peroxidase 4 pathway. Overall, our findings highlight the potential use of *Lycium barbarum* glycopeptide as a neuroprotective agent targeting both ferroptosis and oxidation to decrease ischemic brain damage.

## Introduction

Recent studies have highlighted the significance of ferroptosis in ischemic brain injury (Dávalos et al., 1994; Selim and Ratan, 2004). Ferroptosis is characterized by intracellular iron overload, which triggers lipid peroxidation and excessive generation of reactive oxygen species (ROS) (Rodrigo et al., 2013; Hua et al., 2025; Yang et al., 2025). These ROS, in turn, damage neurons and glia (Cui et al., 2021; Sun et al., 2022), leading to impaired brain function (Shvedova et al., 2021). Iron accumulation has also been reported in the aging brain, as well as in patients with Huntington’s, Parkinson’s, and Alzheimer’s diseases (Morris et al., 2018; Uranga and Salvador, 2018). Therefore, restoring homeostasis of intracellular iron levels is a pivotal strategy not only for the treatment of a stroke but also for other diseases that affect the brain.

Intracellular iron levels are regulated in two main ways. Firstly, the regulation of proteins that control influx of iron ions, such as transferrin receptor 1 (TFR1) and divalent metal transporter 1 (DMT1), and efflux of iron ions, such as ferroportin 1 (FPN1), is essential (Yanatori and Kishi, 2019; Liu et al., 2020). Secondly, the modulation of proteins involved in lipid oxidation and antioxidation is also crucial. The System Xc-(cystine-glutamate transporter system)-GSH-GPX4 pathway plays an important role in inhibiting oxidation and ferroptosis (Fang et al., 2022). System Xc- consists of two subunits: solute carrier family 7 member 11 (SLC7A11) and solute carrier family 3 member 2. Glutathione peroxidase 4 (GPX4) plays a pivotal role in preventing lipid peroxidation (Friedmann Angeli et al., 2014). GPX4 can only function enzymatically upon receiving electrons from glutathione (GSH) (Philpott and Ryu, 2014). Conversely, long-chain acyl-CoA synthetase 4 (ACSL4) promotes ROS generation and contributes to ferroptosis (Kagan et al., 2017). Inhibition of ACSL4 mitigates ferroptosis to facilitate neurological recovery following stroke (Chen et al., 2021). Cerebral ischemia induces downregulation of FPN1 and GPX4 and upregulation of TFR1, DMT1, and ACSL4 (Ingrassia et al., 2019; Cui et al., 2021).

Dixon and colleagues identified Erastin, a small molecule compound with a unique ability to selectively induce iron-dependent cell death (Dixon et al., 2012). In contrast to Erastin, Liproxstatin-1 (Lip-1) has recently emerged as a small molecule compound with antioxidant and anti-ferroptotic properties.

Many Chinese medicines have antioxidant and anti-inflammatory effects and have been proposed as anti-ferroptotic treatments following stroke (Lou et al., 2022). *Lycium barbarum* glycopeptide (LbGp) is derived from *Lycium barbarum* polysaccharides (LBPs), which are extracted from wolfberry, a plant used in Chinese medicine. LbGp is a glycoprotein with a molecular weight of 88 kDa that has robust immunomodulatory and antioxidant capabilities, as demonstrated in previous studies (Huang et al., 2022; Kong et al., 2024). However, whether LbGp mitigates neuronal ferroptosis and improves brain function following ischemic stroke remains unknown. Based on the characteristics of LbGp, we hypothesized that it may protect neurons from ferroptosis because of its strong antioxidative effects. In this study, we used the ferroptosis inhibitor Lip-1 and the ferroptosis inducer Erastin as controls to investigate the effects of LbGp on neuronal ferroptosis in a rat model of cerebral ischemia and explore the underlying mechanisms.

## Methods

### Animals

Male Sprague–Dawley (SD) rats aged 6–8 weeks and weighing between 200 and 250 g were used to avoid the neuroprotective effects of estrogen in female rats (Liu and Mcccllough, 2011). The rats were obtained from Liaoning Changsheng Biotechnology Co., Ltd. (license No. SCXK (Liao) 2020-0001) and were subsequently housed within the Animal Center of Jinan University School of Medicine in a specific pathogen-free environment with a constant temperature range of 23–25°C and a fixed light cycle of 12 hours of light and 12 hours of darkness. The rats were provided with *ad libitum* access to both food and water. The use of animals in this research complied with the regulations set by the Jinan University Experimental Animal Ethics Committee (ACUC-20220114-010, approval date: September 12, 2022). All experiments were designed and reported according to the Animal Research: Reporting of *In Vivo* Experiments (ARRIVE) guidelines (Percie du Sert et al., 2020) and conducted in strict accordance with the National Institutes of Health Guide for the Care and Use of Laboratory Animals (8^th^ ed., National Research Council, 2011).

### Middle cerebral artery occlusion

The intraluminal filament method was used to establish a rat model of middle cerebral artery occlusion (MCAO), as described by Longa et al. (1989), with one modification. Briefly, anesthesia with induced by inhalation of 5% isoflurane (RWD, R510-22-16, Shenzhen, China) and maintained with 2%–2.5% isoflurane, along with 1.0 L/min of oxygen and 1.0 L/min of nitrous oxide (Li et al., 2017). Once anesthetized, the rats were positioned supine on the surgical table. The surgical area was sterilized to maintain aseptic conditions. A midline sagittal incision was carefully made in the anterior neck region. Then, the right common carotid artery (CCA), external carotid artery (ECA), and internal carotid artery (ICA) were exposed. The distal end of the ECA was ligated, and a ligature was applied to temporarily occlude blood flow in the CCA. A hole was created in the ECA, and a filament was carefully advanced through the ECA into the ICA until it reached the branching point of the middle cerebral artery (MCA) within the brain, where a slight resistance was felt. The filament was secured at the ECA using a suture thread. Subsequently, the ligature on the CCA was released to restore blood flow, and the surgical incision was sutured. The rat was returned to its cage, and, 1.5 hours later, was anesthetized again. The surgical site was reopened, and the filament was gently removed. The incision was then sutured, and the animal was placed back in its cage to recover in an environment maintained at 37°C.

The criteria for successful establishment of the MCAO model were neurological function scores of 7–10 and infarction area comprising 20%–40% of the whole brain, as detected by triphenyltetrazolium chloride (TTC) staining and magnetic resonance imaging (MRI) (Fluri et al., 2015).

### Study design

**Figure 1:**
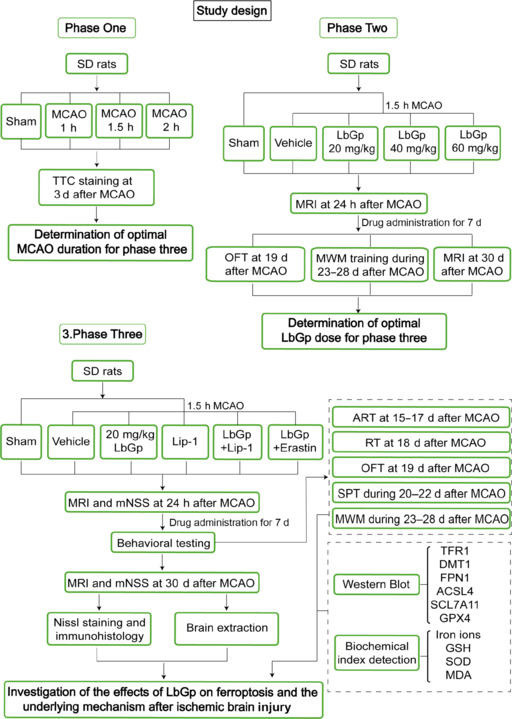
Study design. ACSL4: Long-chain acyl-CoA synthetase 4; ART: adhesive removal test; DMT1: divalent metal transporter; FPN1: ferroportin 1; GPX4: glutathione peroxidase 4; GSH: glutathione; LbGp: *Lycium barbarum* glycopeptide; MCAO: middle cerebral artery occlusion; MDA: malondialdehyde; mNSS: modified neurological severity scores; MRI: magnetic resonance imaging; MWM: Morris water maze; OFT: open field test; RT: rotarod test; SCL7A11: solute carrier family 7 member 11; SD: Sprague-Dawley; SOD: superoxide dismutase; SPT: sucrose preference test; TFR1: transferrin receptor 1; TTC: 2,3,5-triphenyltetrazolium chloride.

This study comprised three distinct phases (**Figure 1**). In phase one, we investigated the correlation between the duration of cerebral ischemia in rats and the degree of brain damage, aiming to determine the optimal ischemia duration for the formal experiment performed in phase three. Rats were randomly assigned to the following four groups: the sham group (cervical skin was cut and the CCA, ICA, and ECA were exposed, but MCAO was not performed; denoted as 0 hours), the 1-hour MCAO group, the 1.5-hour MCAO group, and the 2-hour MCAO group. Each group initially consisted of 10 rats. The infarct volume was determined by TTC staining of the brains from four MCAO rats from each group.

In phase two, the aim was to determine the optimal LbGp dosage by measuring the infarction area using MRI and behavioral assessments. Rats were randomly allocated into five groups: the sham group, the vehicle control group, the MCAO + LbGp (20 mg/kg) group, the MCAO + LbGp (40 mg/kg) group, and the MCAO + LbGp (60 mg/kg) group. Each group comprised six rats.

In phase three, we investigated the effects of LbGp administered at a dosage of 20 mg/kg on pathology, behavior, and ferroptosis MCAO (1.5 hours) rats. Rats were randomly assigned to six groups: the sham group, the vehicle control group (MCAO only group), the MCAO + LbGp (20 mg/kg) group, the MCAO + Lip-1 (5 mg/kg) group, the MCAO + LbGp (20 mg/kg) + Lip-1 (5 mg/kg) group, and the MCAO + LbGp (20 mg/kg) + Erastin (25 mg/kg) group. Initially, each experimental group consisted of 10 rats, similar to Deborah et al. (2012) and Wang et al. (2023). Following MCAO induction, some rats were excluded because they did not meet the criteria (the infarct volume should be between 20% and 40% of the total brain volume). Additionally, some rats died during or after the MCAO procedure. Ultimately, six rats in each group underwent behavioral assessments, pathological examinations, and protein analyses.

### *Lycium barbarum* glycopeptide preparation

The LbGp utilized in this study was generously provided by the Academician Workstation of Ningxia Zhongning Goji College. The preparation of LbGp involved two distinct phases. In the initial phase, LBPs were extracted from dried goji berries (Yuan et al., 2016). In the second stage, LBPs underwent purification, elution, dialysis, and concentration through the use of DEAE-Cellulose column chromatography and Sephadex G100 gel filtration chromatography (Peng and Tian, 2001). The LbGp that was ultimately obtained exhibited a purity level of 92.5% ± 3.5%. The purified LbGp was identified as having a molecular weight of 88 kDa and a sugar content of 70%. The sugar composition of LbGp is as follows: Ara:Gal:Glc = 2.5:1.0:1.0 (molar ratio). Generally, LbGp contains nearly all naturally occurring amino acids, with a protein content of approximately 30%.

### Drug administration

LbGp was dissolved in normal saline and administered via oral gavage. Lip-1 (Selleck, Houston, TX, USA, S7699) was dissolved in anhydrous ethanol, and Erastin (TargetMol, Boston, MA, USA, T1765) was dissolved in 10% DMSO. Both Lip-1 and Erastin were administered via intraperitoneal injection. In phases two and three, animals received LbGp for 7 consecutive days. The medication period was determined based on the results of our previous study, which showed that administration of LBP for 7 consecutive days prevents neural loss in the hippocampus after transient cerebral ischemia in rats (Shi et al., 2017). In addition, we previously observed a reduction in both infarct size and neurological deficit score in MCAO mice after LBP pre-treatment for 7 days (Yang et al., 2012; Wang et al., 2014). In this study, we began LbGp treatment 24 hours after ischemia, immediately following the first MRI scan.

### Triphenyltetrazolium chloride staining

On the third day following MCAO in phase one, rats were anesthetized with 5% isoflurane gas and decapitated, and their brains were promptly removed. Subsequently, the whole brain was rapidly frozen at –80°C for 2 minutes and then sliced into five to six approximately 2-mm-thick coronal brain sections. These brain sections were immersed in a 1% 2,3,5-triphenyltetrazolium chloride solution (Sigma, St. Louis, MO, USA, Cat# BCBW4269) in a dark environment at 37°C for 30 minutes. Then, images were taken of the stained brain sections.

### Brain tissue processing and sectioning

Rats were anesthetized with 5% isoflurane gas and then perfused through the heart with a 4% paraformaldehyde solution for a duration of 20 minutes. A craniotomy was performed, and the brain was carefully extracted and placed in 4% paraformaldehyde for 24 hours. The fixed brains were subsequently dehydrated in a series of sucrose solutions of increasing concentrations (10%, 20%, and 30%). Following dehydration, the brains were sliced into 4-mm-thick coronal sections containing the infarcted area. These coronal sections were then sliced at a thickness of 20 μm using a cryostat (Leica, Weztlar, Germany). After sectioning, the glass slides containing the brain sections were air-dried and then stored in slide boxes in a freezer at –40°C for long-term preservation.

### Nissl staining

To visualize and quantify changes in the brain structures post-ischemia, we used a Nissl staining kit (Solarbio, Beijing, China, G1430) according to the manufacturer’s instructions. Briefly, the slide-mounted sections were defatted in a 1:1 solution of ethanol and chloroform overnight, hydrated in an ethanol series (100%, 95%, 80%, and 70%, for 2 minutes each), Nissl stained at 56°C in an oven for 1 hour, rinsed with deionized water, differentiated for a few seconds to 2 minutes, dehydrated in an ethanol series (70%, 80%, 95%, and 100%, for 3 minutes each), cleared with xylene twice (for 3 minutes each time), and finally sealed with neutral gum.

### Immunofluorescence staining

In phase three, we examined the effect of LbGp on inflammation by immunofluorescence staining using an anti- ionized calcium-binding adapter molecule 1 (Iba1) antibody. First, to block non-specific binding of antibodies, brain sections were treated with 0.3% Triton X-100 (Sigma) in 0.01 M phosphate-buffered saline (PBS) containing 1% bovine serum albumin (Sigma) and 5% goat serum (Sigma) at room temperature for 2 hours. Subsequently, primary antibody staining was performed using a rabbit anti-rat Iba1 antibody (1:1000, FUJIFILM, Tokyo, Japan, Ca# 019-19741, RRID: AB_8839504) at 4°C. After three washes with PBS for 10 minutes each, the sections were incubated with a donkey anti-rabbit secondary antibody labeled with Alexa Fluor 546 (1:5000, Thermo Fisher Scientific, Waltham, MA, USA, Ca# A16024, RRID: AB_2534698) at room temperature for 2 hours. Following three additional rinses with PBS, the sections were mounted with an anti-fade mounting medium containing 4′,6-diamidino-2-phenylindole (1:1000, Abcam, Cambridge, UK, Ca# ab285390) for nuclear counterstaining.

### Behavioral tests

The order of the following six behavioral tests was arranged based on the physiological condition of the rats, as well as the complexity and difficulty of the tasks for the rats following stroke. The behavior tests were based on Schaar et al. (2010).

#### Neurological function assessment

To evaluate effects of LbGp on the neurological function of rats post-ischemia, we employed the modified neurological severity score (mNSS) developed by Chen et al. (2001) and Shi et al. (2021) on days 1 and 30 after MCAO. The mNSS assesses sensory, motor, reflex, balance, and overall neurological functions and is scored on a scale ranging from 0 to 18 points. A score of 0 indicates normal function, scores from 1 to 6 indicate mild impairment, scores from 7 to 12 indicate moderate impairment, and scores from 13 to 18 indicate severe impairment.

#### Adhesive removal test

The adhesive removal test (Bu et al., 2024) was conducted to assess sensory and motor function in rats. On days 15, 16, and 17 post-MCAO, the animals were placed in the experimental cage for 2 minutes to adapt to the test environment. On the day of the test, small squares of adhesive tape (approximately 6 mm per side) were attached to the forepaws of the rats. The time taken by the animals to remove the tape from their forepaws was recorded. The average time was calculated from three consecutive tests. The interval between each test was 30 minutes.

#### Rotarod test

To evaluate the animals’ motor coordination, balance, and endurance, a rotarod test (RT) (Liu et al., 2023) was conducted on day 18 post-MCAO. Prior to the test, rats underwent a 5-minute training session on a circular rod set at a constant speed of 4 r/min. In the formal experiment, the speed of the rotarod gradually increased, starting from 4 r/min and reaching a maximum of 40 r/min over time. The time that rats spent on the rod before they fell off was recorded. Each animal underwent three repetitions of the rotarod test, with a 15-minute interval between each repetition. The average duration on the rod was calculated from the three consecutive tests.

#### Open field test

The open field test (OFT) was administered on day 19 post-MCAO to evaluate anxiety and exploratory behavior in rats. The test was conducted in an open box measuring 1 m × 1 m, equipped with a computer tracking system. The box was divided into peripheral and central zones. To acclimatize the animals to the testing environment, they were placed in the testing room overnight the day before the test. The rats were placed in the open box to explore and were allowed to move freely for a duration of 10 minutes.

#### Sucrose preference test

The sucrose preference test was conducted 20–22 days post-MCAO to assess the animals’ preference for sweetness and detect depression-like symptoms. Testing began every day at 9:00 p.m. Bottles containing 2% sucrose water or pure water were weighed separately. The weights of the bottles were measured again at 9:00 AM the following morning. This procedure was conducted continuously for three days. Both the sucrose water and pure water were freshly refilled, and the positions of the two bottles were switched, each day. The sucrose preference was calculated using the following formula: Sucrose liquid consumption (g)/(pure water consumption (g) + sucrose liquid consumption (g)). The average preference over the three days was used for analysis and comparison.

#### Morris water maze

A Morris water maze (MWM) test was performed on days 23–28 post-ischemia to assess spatial learning and memory in rodents. The MWM apparatus consists of a circular metal pool with a diameter of 150 cm and a height of 55 cm, partitioned into four quadrants (southwest, southeast, northwest, and northeast). One of these quadrants houses an escape platform 10 cm in diameter and 21 cm in height, positioned at a depth of 1–2 cm beneath the water surface. The water temperature was consistently maintained at 26 ± 1°C throughout the duration of the experiment.

Noldus EthoVision software (Noldus, Wageningen, Netherlands) was utilized to precisely track and record various parameters pertaining to the animals’ performance, including time taken to locate the platform, swimming path, speed, and distance. The experiment spanned a total of 6 days. Over the initial 5 days, the animals underwent a training phase. Two training sessions were conducted each day, with a 30-minute interval between sessions. During each session, the rodents were placed sequentially in each of the four quadrants, initially facing the wall of the pool. In cases where a rodent failed to locate the platform within 1 minute, the experimenter provided guidance using a thin rod, and the escape latency was recorded as 60 seconds. A 15-second resting period on the platform was allowed between quadrant switches. On the sixth day of the experiment, the submerged platform was removed, and the animals were allowed to swim freely for 60 seconds. During this probe trial, the swimming trajectories and the time taken by each rodent to locate the platform (escape latency) were recorded.

### Magnetic resonance imaging

MRI scans were performed 24 hours and 30 days following MCAO in phase two and phase three using a 9.4 T small animal magnetic resonance imaging system (Bruker, Billerica, MA, USA). The scanning parameters were configured as follows: relaxation-enhanced (RARE) sequence capturing T2-weighted images, with a repetition time (TR) of 6000 ms, echo spacing (ESP) of 10.667 ms, field of view (FOV) measuring 23 × 23 mm^2^, flip angle of 90°, refocusing angle of 180°, bandwidth of 40 kHz, echo train length (ETL) of 8, k-0 = 3, echo time (TE) of 32 ms, averages = 2, slices = 28, and slice thickness = 1.0 mm.

### Western blotting

Western blotting was carried out at 30 days post-MCAO after the rats had completed all of the behavioral tests. The animals were anesthetized with 5% isoflurane gas and then perfused with physiological saline through the heart to remove blood. Brains were quickly removed on ice, and brain tissue from the ischemic penumbra of the cortex (located 2 mm beyond infarction area) was dissected. The brain tissues were processed using routine procedures for Western blotting, including protein extraction using RIPA lysis buffer and sonication; protein quantification using a BCA protein analysis kit; protein separation by sodium dodecyl sulfate-polyacrylamide gel electrophoresis (SDS-PAGE, Beyotime, Shanghai, China, P0012A); protein transfer to a polyvinylidene difluoride membrane (Merck KGaA, Darmstadt, Germany, 03010040001); membrane blocking with 5% non-fat milk for 2 hours; and primary antibody incubation at 4°C overnight. Rabbit primary antibodies to the following proteins were used: GPX4 (1:2000, Abcam, Cat# ab125066, RRID: AB_10973901), ACSL4 (1:2000, GeneTex, Irvine, CA, USA, Cat# GTX635616, RRID: AB_2888541), ferroptin-1 (1:2000, GeneTex, Cat# GTX554821, RRID: AB_2864856), DMT1 (1:2000, GeneTex, Cat# GTX64686, RRID: AB_2199454), SLC7A11(1:2000, Cell Signaling Technology, Danvers, MA, USA, Cat# 98051, RRID: AB_2800296), and TFR1 (1:2000, Abcam, Cat# ab129177, RRID: AB_11142009). The membranes were washed three times with 0.1% TBST for 10 minutes each time and then incubated with goat anti-rabbit IgG (Abcam, Cat# ab150077, RRID: AB_2630356, 1:5000) at room temperature for 2 hours. Following another three washes with 0.1% TBST for 10 minutes each time, the protein bands were visualized using a gel imaging system (Bio-Rad, Hercules, CA, USA). Finally, ImageJ 1.40 software (NIH, Bethesda, MD, USA) was used to quantify the grayscale values of each band relative to one of the internal controls: GAPDH (Abcam, Ca# ab8245, RRID: AB_2107448) or tubulin (Abcam, Ca# ab7291, RRID: AB_2241126).

### Measurement of glutathione, superoxide dismutase, malondialdehyde, and iron content

The information of all kits used to measure glutathione (GSH), superoxide dismutase (SOD), malondialdehyde (MDA), and iron content in the ischemic penumbra tissue is as follows: GSH (Solarbio, BC1175, Beijing, China), SOD (Solarbio, BC0175), MDA (Solarbio, BC0025), and iron ions (Nanjing Jiancheng Bioengineering Institute, A039-2-1, Nanjing, China). These substances were measured using the colorimetric method (Xiao et al., 2022) on day 30 after MCAO. All procedures for these assays were strictly adhered to the instructions provided with the procured assay kits.

### Measurement of infarct volumes in middle cerebral artery occlusion rats by triphenyltetrazolium chloride staining and magnetic resonance imaging

Each group consisted of four rats for TTC staining in phase one. Images of the brain infarct areas were captured using ImageJ software. To account for the impact of edema on infarct volume, we adopted the formula proposed by Loihl et al. (1999) to calculate infarct volume: Infarct volume (%) = (volume of the contralateral brain – volume of the unimpaired ipsilateral brain)/Total brain volume ×100. This approach helps accurately assess the infarct size while considering the presence of edema.

The infarct volume in six rats from each group was compared based on MRI images at 24 hours and 30 days after MCAO. Fifteen MRI scan images of each animal were analyzed using ImageJ. The infarct volume based on the MRI scans was measured and calculated using the same formula as that used for the TTC staining images.

### Quantification of Nissl-positive and ionized calcium-binding adapter molecule 1-positive cells

Each group for cell count analysis consisted of three animals. From each animal, three brain sections separated by 600-μm intervals were selected for Nissl staining. The stained brain sections were imaged using a bright-field microscope (Leica). A rectangular region measuring 0.5 mm × 1.5 mm in the penumbra of the ischemic cortex was chosen for analysis. For quantitative analysis of immunofluorescence staining, the same regions as those used for Nissl staining were selected, and the brain sections were imaged under an upright fluorescence microscope (Leica). Neuronal cells with positive Nissl staining and glial cells with positive Iba1 immunofluorescence staining were counted using ImageJ software.

### Statistical analysis

No statistical methods were used to predetermine sample sizes; however, our sample sizes are similar to those reported in previous publications (Deborah et al., 2012; Wang et al., 2023). Statistical analysis was performed and graphs were drawn using GraphPad Prism 8 software (GraphPad Software, San Diego, CA, USA, www.graphpad.com). ImageJ (1.40) and Image-Pro Plus 6.0 (Media Cybernetics, Inc, Rockville, MD, USA) were used for data analysis of Western blot and immunofluorescence images. The data are presented as mean ± standard deviation. Multiple group comparisons were conducted using one-way analysis of variance followed by Tukey’s honestly significant difference (HSD) test for post hoc analysis of differences between groups. For comparisons between two independent samples, the independent samples *t*-test was used. *P* < 0.05 was considered to be statistically significant.

## Results

### Optimization of occlusion duration for comparing survival and infarction size post-ischemia

**Figure 2:**
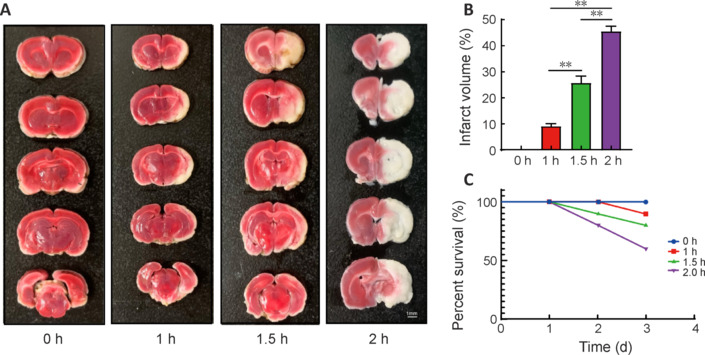
Impact of various ischemia durations on rat brain infarct volume and survival rates. (A) Representative images of brain slices (2 mm) stained with triphenyltetrazolium chloride on the 3^rd^ day post-middle cerebral artery occlusion. The area in red indicates normal brain tissue, while the area in white represents infarcted tissue. (B) Different durations of ischemia in rats resulted in varying sizes of brain infarct volumes. The results are presented as mean ± SD (*n* = 4). (C) Survival rates of rats subjected to different durations of ischemia within 3 days after middle cerebral artery occlusion. **P < 0.01.

On day 3 following MCAO in phase one, TTC staining showed various infarct areas in different groups (**Figure 2A**). Significant differences in infarct volumes were observed among the three groups (*P* < 0.01). All three experimental groups also exhibited significant differences compared with the sham group (*P* < 0.01; **Figure 2B**). Furthermore, the survival rate of MCAO rats on the first day following ischemia was 100% for all groups (**Figure 2C**). However, on the second day, the survival rate remained at 100% for the sham group and the 1-hour MCAO group, while it decreased to 90% for the 1.5-hour MCAO group and 80% for the 2-hour MCAO group. By the third day, no rats had died in the sham group, but the survival rate decreased to 90% for the 1-hour ischemia group, 80% for the 1.5-hour ischemia group, and only 60% for the 2-hour ischemia group.

Based on the results from phase one, 1.5 hours was selected as the optimum duration of MCAO. The infarct size in these rats met the criteria (20%–40%) that we set for the formal experiment, while the 1-hour and 2-hour MCAO rats did not meet this metric. Additionally, the 1.5-hour MCAO rats had a higher survival rate than the 2-hour MCAO rats.

### Optimization of *Lycium barbarum* glycopeptide dose for treating middle cerebral artery occlusion rats post-ischemia

**Figure 3:**
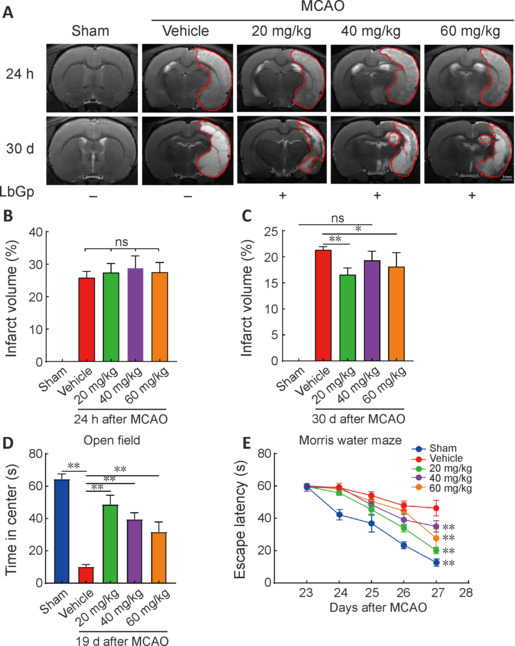
Effects of different LbGp concentrations on brain infarct volume and behaviors in MCAO rats. (A) Representative MRI images displaying infarcted areas of rat brains in the different dose groups at 24 hours and 30 days post-MCAO. The infarcted areas are indicated by red circles. “–” indicates no LbGp administration, and “+” indicates LbGp administration. (B, C) Infarct volumes in each group as measured by MRI at 24 hours and 30 days post-MCAO. (D) OFT results showing exploration time in the central area for the different dose groups. (E) MWM results demonstrating escape latency in the different dose groups. (B–E) The results are presented as mean ± SD (*n* = 6), *P < 0.05, **P < 0.01. LbGp: *Lycium barbarum* glycopeptide; MCAO: middle cerebral artery occlusion; MRI: magnetic resonance imaging; MWM: Morris water maze; OFT: open field test.

In phase two, we first selected an LbGp dose based on the infarct volume as determined by MRI scans. The MRI scans showed various infarct areas (grey areas encircled by red lines) in the different groups at 24 hours and 30 days post-MCAO (**Figure 3A**). Quantitative analysis demonstrated that there were no statistically significant differences in infarct volumes among the vehicle, 20 mg/kg LbGp, 40 mg/kg LbGp, and 60 mg/kg LbGp groups at 24 hours after MCAO (*P* > 0.05; **Figure 3B**). However, all these groups exhibited significant differences compared with the sham group (*P* < 0.01). On day 30 post-MCAO (**Figure 3C**), compared with the vehicle group, the 20 mg/kg LbGp and 60 mg/kg LbGp groups (*P* < 0.05) showed a significant reduction in infarct size, while the 40 mg/kg LbGp group showed no significant difference (*P* > 0.05).

To determine the optimal dose of LbGp for the treatment of MCAO rats, we also conducted the OFT and MWM The OFT results (**Figure 3D**) indicated that the exploration time in the center area for the vehicle rats was significantly decreased compared with the sham group (*P* < 0.01). This duration was also decreased compared to groups treated with LbGp at concentrations of 20, 40, and 60 mg/kg (all *P* < 0.01). Notably, the 20 mg/kg LbGp group spent more time in the central area compared with the 40 mg/kg (*P* < 0.05) and 60 mg/kg LbGp groups (*P* < 0.01).

Subsequently, from days 23 to 28 post-MCAO, we assessed the spatial learning and memory of rats treated with different LbGp concentrations, using the MWM test. The MWM results (**Figure 3E**) revealed that, on the first day of training, there were no significant changes in escape latency (time to find the platform) among the five groups (all took 60 seconds). On the second day, the sham group demonstrated a significant decrease in escape latency compared with other four groups (*P* < 0.05). On the third day, a significant difference between the 20 mg/kg LbGp and vehicle groups appeared (*P* < 0.01). However, no significant difference was observed between the 20 mg/kg LbGp and the 40 mg/kg LbGp and 60 mg/kg LbGp groups, even though the 20 mg/kg LbGp group had a longer latency than the sham group (*P* < 0.05). On the fourth day, as the training continued, the escape latency of the 20 mg/kg LbGp and 40 mg/kg LbGp groups was significantly reduced compared with the vehicle group (*P* < 0.01). By the fifth day, the escape latency of the 20 mg/kg LbGp, 40 mg/kg LbGp, and 60 mg/kg LbGp groups showed significant differences compared with the vehicle group (*P* < 0.01).

Based on the results obtained from phase two, the administration of 20 mg/kg LbGp not only reduced infarct volume but also improved learning and memory. This concentration was considered the ideal dose for in the experiments in phase three.

### Treatment with *Lycium barbarum* glycopeptide and Liproxstatin-1 reduces infarct volume and neuronal loss in middle cerebral artery occlusion rats

**Figure 4:**
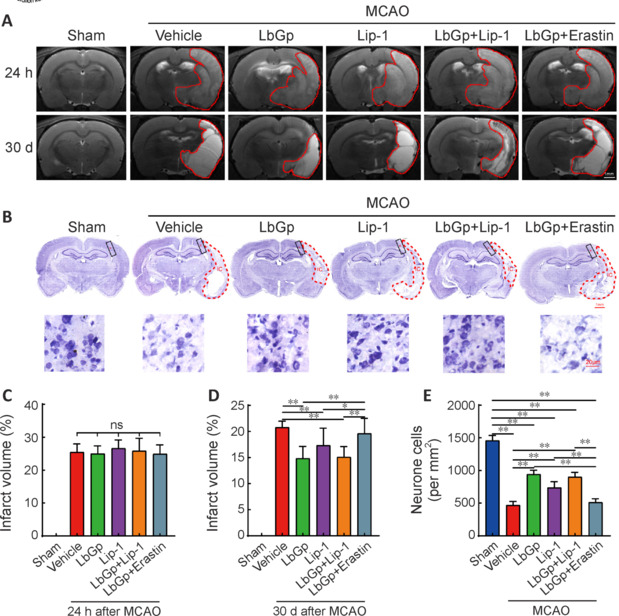
Impact of LbGp and Lip-1 on cerebral infarct volume and neuron count. (A) MRI images illustrating cerebral infarct areas (circled in red) in rats from each group at 24 hours and 30 days post-MCAO. (B) Representative Nissl staining images, with the ischemic core area outlined by red dashed lines and the adjacent penumbral region outlined by rectangles measuring 500 μm × 1500 μm. (C and D) Percentage change in the cerebral infarct area in rats, as measured by MRI, at 24 and 30 days post-MCAO, respectively. The results are presented as mean ± SD (*n* = 6). (E) Numbers of neuronal cells in the penumbral region, as assessed by Nissl staining. The results are presented as mean ± SD (*n* = 3), *P < 0.05, **P < 0.01. Erastin: A ferroptosis activator; LbGp: *Lycium barbarum* glycopeptide; Lip-1: liproxstatin-1 (a ferroptosis inhibitor); MCAO: middle cerebral artery occlusion; MRI: magnetic resonance imaging.

In phase three, we first aimed to investigate the effect of LbGp on infarct area. MRI scans (**Figure 4A** and **C**) revealed that there were no significant differences in infarct volume (between 25%–30%) among the groups 24 hours after MCAO (*P* > 0.05), except for the sham group (an infarct volume of 0). However, at 30 days post-MCAO, the LbGp, Lip-1, and LbGp + Lip-1 groups exhibited a significant reduction in infarct volumes compared with the vehicle group (all *P* < 0.05; **Figure 4A** and **D**). Notably, when Erastin was introduced, the infarct volume increased, showing significant differences compared with the LbGp group (*P* < 0.01), the Lip-1 group (*P* < 0.05), and the LbGp + Lip-1 group (*P* < 0.01).

The neurons in the sham group exhibited robust Nissl staining (**Figure 4B** and **E**). In contrast, the vehicle group displayed lighter Nissl staining in the ischemic penumbra, accompanied by a reduced cell count. However, the LbGp, Lip-1, and LbGp + Lip-1 groups exhibited more neurons with deeper Nissl staining than the vehicle group (all *P* < 0.01). Treatment with Erastin decreased the number of neurons to significantly lower than the three drug-treated groups (*P* < 0.01 in all cases).

### Treatment with *Lycium barbarum* glycopeptide and Liproxstatin-1 improves neuronal function and behaviors in middle cerebral artery occlusion rats

**Figure 5:**
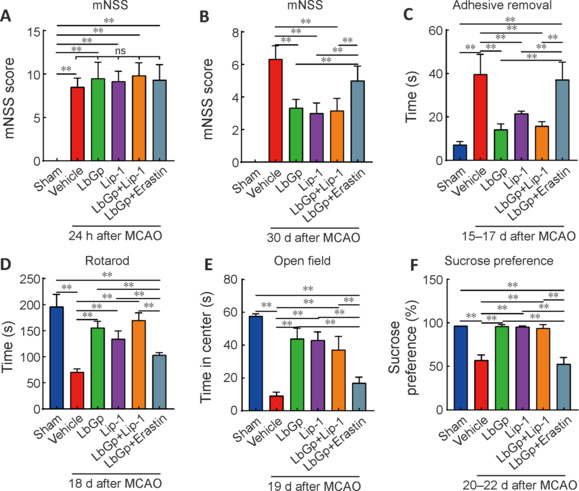
Impact of LbGp and Lip-1 on neurological function and behaviors in MCAO rats. (A, B) mNSS scores from the different groups at 24 hours and 30 days post-MCAO. (C) Time taken for rats to remove tape from the left forelimb in the adhesive removal test. (D) Fall latencies of the rats in the rotarod test. (E) Time spent in the center zone in the open field test. (F) Sucrose preference percentage in rats from the various groups. The results are presented as mean ± SD (*n* = 6), **P < 0.01. Erastin: A ferroptosis activator; LbGp: *Lycium barbarum* glycopeptide; Lip-1: liproxstatin-1 (a ferroptosis inhibitor); MCAO: middle cerebral artery occlusion; mNSS: modified neurological severity scores.

Next, we investigated the effect of LbGp on neurogical function. At 24 hours after MCAO, all groups exhibited severe neurological deficits, with mNSS scores ranging from 8 to 12, which was significantly different than the sham group (score of 0) (all *P* < 0.01; **Figure 5A**). The rats were then treated with different drugs for 7 days, and neurological function was reevaluated at 30 days post-MCAO. mNSS in the sham group remained at 0, while the vehicle group’s score increased significantly. In contrast, the LbGp, Lip-1 group, and LbGp + Lip-1 group exhibited significantly reduced neurological function scores compared with the vehicle group (all *P* < 0.01; **Figure 5B**). However, administration of Erastin significantly increased the mNSS score to a level comparable to that in the vehicle group.

Sensory function was assessed by the adhesive removal test. The sham group rats took 7.1 ± 1.4 seconds to remove the adhesive from their left limbs (**Figure 5C**). In contrast, the vehicle group rats exhibited significantly longer adhesive removal times compared with the sham group (*P* < 0.01). However, the LbGp, Lip-1, and LbGp + Lip-1 groups exhibited significantly reduced adhesive removal time compared with the vehicle group (all *P* < 0.01). The administration of Erastin significantly increased the removal time compared with the LbGp, Lip-1, and LbGp + Lip-1 groups (all *P* < 0.01).

Next, the rotarod test was conducted to assess motor function. The vehicle group rats spent significantly less time on the rod than the sham group (*P* < 0.01). However, the LbGp, Lip-1, and LbGp + Lip-1 groups exhibited significantly increased time on the rod compared with the vehicle group (all *P* < 0.01). Notably, the effect of LbGp was significantly reversed by treatment with Erastin (**Figure 5D**).

An OFT was employed to evaluate anxiety levels in the rats. The vehicle group rats spent significantly less time exploring the central area than the sham group rats (*P* < 0.01). Rats in the LbGp, Lip-1, and LbGp + Lip-1 groups spent significantly more time exploring the central area compared with rats in the vehicle group (all *P* < 0.01). However, LbGp’s effectiveness was significantly reduced when Erastin was administered (all *P* < 0.01), compared with the three drug-treated groups (**Figure 5E**).

The sucrose preference test was utilized to assess depression-like behavior in MCAO rats. The results from the sucrose preference test (**Figure 5F**) indicated that the sucrose preference levels in the LbGp, Lip-1, and LBGP + Lip-1 groups closely resembled those of the sham group. In contrast, the sucrose preference levels in the vehicle and LbGp + Erastin groups were significantly lower compared with the sham group and the other three drug-treated groups (all *P* < 0.01). These findings suggest that LbGp effectively ameliorates anxiety and depression-like behavior in MCAO rats.

### Treatment with *Lycium barbarum* glycopeptide and Liproxstatin-1 improves spatial learning and memory in middle cerebral artery occlusion rats

**Figure 6:**
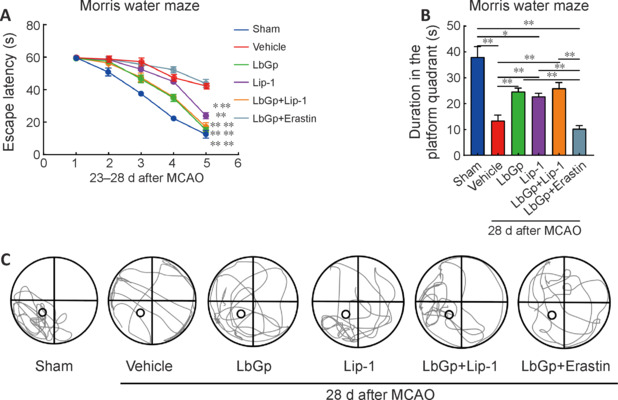
Impact of LbGp and Lip-1 on spatial learning and memory in MCAO rats. (A) The escape latency of rats in the different groups in the first 5 days of MWM training. (B) Time spent by rats in the quadrant with a platform after removing the platform on the 6^th^ day of MWM test in the different groups. (C) Swim paths of rats in the various groups. (A, B) The results are presented as mean ± SD (*n* = 6), *P < 0.05, **P < 0.01; ##*P* < 0.01; &&*P* < 0.01. Erastin: A ferroptosis activator; LbGp: *Lycium barbarum* glycopeptide; Lip-1: liproxstatin-1 (a ferroptosis inhibitor); MCAO: middle cerebral artery occlusion; MWM: Morris water maze.

A Morris water maze test was conducted to evaluate spatial learning and memory in rats (**Figure 6**). On the first day of training, there were no significant differences in escape latency among the groups, with values ranging from 58 to 60 seconds (**Figure 6A**). Starting from the second day, the escape latency of rats in the sham group significantly decreased. While the rats in the other groups also exhibited gradual reductions in escape latency, the rate of decline varied. By the fifth day, the escape latency of the LbGp, Lip-1, and LbGp + Lip-1 groups closely resembled that of the sham group and was significantly shorter than that of the vehicle and LbGp + Erastin groups (all *P* < 0.01). Notably, there was no significant difference between the LbGp and sham groups (*P* > 0.05). However, the escape latency of the Lip-1 group was still significantly longer than that of the sham group (*P* < 0.05).

On the sixth day, the swimming paths of rats were recorded after the submerged platform was removed. Rats in the sham, LbGp, Lip-1, and LbGp + Lip-1 groups spent significantly more time in the target quadrant compared with rats in the vehicle and LbGp + Erastin groups (all *P* < 0.01; **Figure 6B** and **C**). Neither the LbGp group nor the LbGp + Lip-1 group showed a significant difference compared with the sham group (*P* > 0.01). However, the Lip-1 group spent less time in the target quadrant compared with the sham group (*P* < 0.05).

### *Lycium barbarum* glycopeptide and Liproxstatin-1 treatment lower iron ion levels and modulate iron transport protein expression

**Figure 7:**
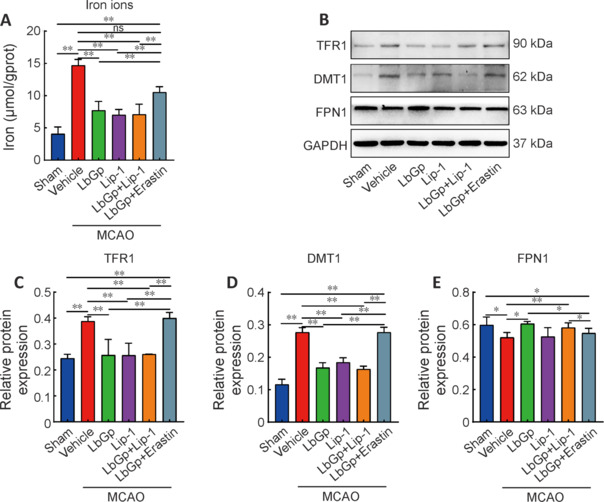
Impact of LbGp and Lip-1 on iron ion content and expression of iron transport proteins in the cortical ischemic penumbra of MCAO rats. (A) Iron ion content in rats from various groups, as measured by the colorimetric method. (B) Western blots of TFR1, DMT1, FPN1. (C–E) Quantitative analysis of TFR1, DMT1, and FPN1 expression in each group. (A, C, D, E) The results are presented as mean ± SD (*n* = 3), *P < 0.05, **P < 0.01. Experimental times: *n* = 3. DMT1: Divalent metal transporter; Erastin: a ferroptosis activator; FPN1: ferroportin 1; LbGp: *Lycium barbarum* glycopeptide; Lip-1: liproxstatin-1 (a ferroptosis inhibitor); MCAO: middle cerebral artery occlusion; TFR1: transferrin receptor 1.

On day 30 after MCAO, iron ion levels in the ischemic penumbra of rats were measured. The results demonstrated the iron ion content was significantly increased in the vehicle group compared with the sham group (*P* < 0.01; **Figure 7A**). However, treatment with LbGp, Lip-1, or LbGp + Lip-1 led to significant reductions in iron ion levels compared with the vehicle group (all *P* < 0.01). Notably, when Erastin was administered, the iron ion levels increased significantly to levels that were not significantly different from those seen in the vehicle group (*P* > 0.05).

Furthermore, we investigated the expression levels of proteins that regulate iron ion transport, specifically focusing on TFR1, DMT1 and FPN1. After MCAO, TFR1 levels (relative protein expression) were significantly increased in the vehicle group compared with the sham group (*P* < 0.01; **Figure 7B** and **C**). However, after treatment, TFR1 expression was significantly decreased in the LbGp, Lip-1, and LbGp + Lip-1 groups compared with the vehicle group (all *P* < 0.01). Conversely, the addition of Erastin led to an increase in TFR1 expression, to a level that was significantly different from those seen in the three drug-treated groups (*P* < 0.01).

The DMT1 expression trends mirrored those of TFR1. After ischemia, DMT1 expression was significantly increased in the vehicle group compared with the sham group (*P* < 0.01; **Figure 7D**). However, DMT1 expression was significantly decreased in the LbGp, Lip-1, and LBGP + Lip-1 groups compared with the vehicle group (all *P* < 0.01). Conversely, the addition of Erastin led to a significant increase in DMT1 expression, to a level very close to that seen in the vehicle group.

In contrast, after MCAO, FPN1 expression levels were decreased in the vehicle group (**Figure 7E**) compared with the sham group (*P* < 0.05). Intriguingly, the Lip-1 group exhibited a similar decrease in FPN1 expression. However, FPN1 levels in the LbGp group and LbGp+Lip-1 group were similar to those in the sham group (*P* < 0.05), which were significantly different compared with those in the vehicle, Lip-1, and LbGp + Erastin groups.

### *Lycium barbarum* glycopeptide and Liproxstatin-1 enhance the expression of antioxidant proteins in the System Xc-GSH-GPX4 pathway while suppressing oxidative protein expression

**Figure 8:**
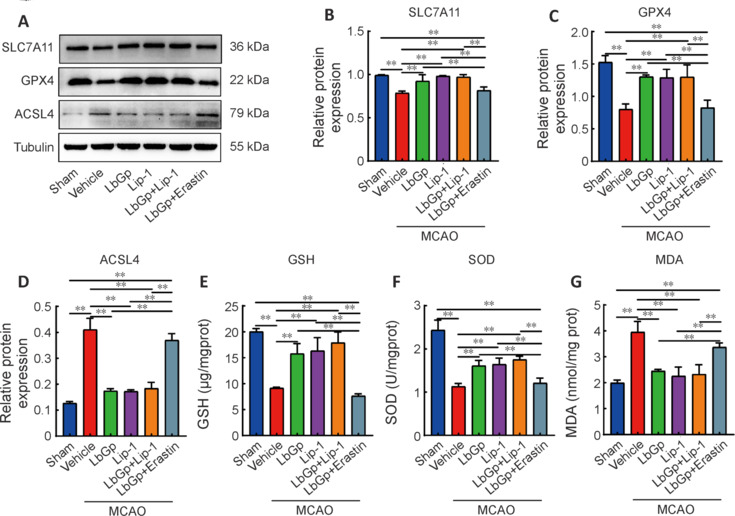
Effects of LbGp and Lip-1 on the expression of critical proteins in antioxidant and oxidative pathways in MCAO rats. (A) Western blot of SLC7A11, GPX4, and ACSL4. (B–D) Quantitative analysis of SLC7A11, GPX4, and ACSL4 protein expression in each group. (E–G) GSH, SOD, and MDA expression levels in the ischemic penumbra of brain tissue from various groups, as measured by the colorimetric method. (B–G) The results are presented as mean ± SD (*n* = 3), **P < 0.01. Experimental times: *n* = 3. ACSL4: Long-chain acyl-CoA synthetase 4; Erastin: a ferroptosis activator; GPX4: glutathione peroxidase 4; GSH: glutathione; LbGp: *Lycium barbarum* glycopeptide; Lip-1: liproxstatin-1 (a ferroptosis inhibitor); MCAO: middle cerebral artery occlusion; MDA: malondialdehyde; SCL7A11: solute carrier family 7 member 11; SOD: superoxide dismutase.

Western blot analysis revealed single protein bands for the antioxidant proteins SLC7A11 and GPX4, as well as the oxidative protein ACSL4 (**Figure 8A**). Quantitative analysis demonstrated that, after MCAO, SLC7A11 expression in the vehicle group was decreased compared with that in the sham group (*P* < 0.01; **Figure 8B**). However, treatment with LbGp, Lip-1, and LbGp + Lip-1 resulted in an increase in SLC7A11 expression compared with the vehicle group (all *P* < 0.01). Upon the addition of Erastin, the SLC7A11 level decreased compared with the three treatment groups (*P* < 0.01).

The GPX4 expression patterns were comparable to those of SLC7A11 (**Figure 8C**). After MCAO, the expression of GPX4 was decreased in the vehicle group compared with the sham group (*P* < 0.01). However, in the LbGp, Lip-1, and LbGp + Lip-1 groups, GPX4 expression was significantly increased compared with the vehicle group (all *P* < 0.01). After the addition of Erastin, GPX4 expression was decreased significantly compared with the LbGp, Lip-1, and LbGp + Lip-1 groups (all *P* < 0.01).

Conversely, after MCAO, ACSL4 expression was increased in the vehicle group compared with the sham group (*P* < 0.01; **Figure 8D**). However, ACSL4 expression in the LbGp, Lip-1, and LbGp + Lip-1 groups was significantly lower than in the vehicle group (*P* < 0.01). Upon the addition of Erastin, ACSL4 expression was significantly increased (*P* < 0.01) compared with the three drug-treated groups.

The GSH expression patterns were comparable to those of GPX4. After MCAO, GSH expression was decreased in the vehicle group (*P* < 0.01) compared with the sham group (**Figure 8E**). However, GSH expression in the LbGp, Lip-1, and LbGp + Lip-1 groups was significantly increased compared with the vehicle group (*P* < 0.01). Upon the addition of Erastin, GSH levels were significantly decreased compared with the LbGp, Lip-1, and LbGp + Lip-1 groups (all *P* < 0.01).

SOD is an antioxidant enzyme responsible for clearing superoxide radicals from cells. After MCAO, the SOD levels in the vehicle group were significantly lower than those in the sham group (*P* < 0.01; **Figure 8F**). However, in the LbGp, Lip-1, and LbGp + Lip-1 groups, SOD levels were significantly increased (*P* < 0.01) compared with the vehicle group. Nevertheless, with the addition of Erastin, SOD levels were significantly decreased compared with the LbGp, Lip-1, and LbGp + Lip-1 groups (all *P* < 0.01).

MDA is a product of lipid peroxidation, and its levels are proportional to the generation of free radicals. After MCAO, the MDA content in the vehicle group was increased significantly (**Figure 8G**) compared with the sham group (*P* < 0.01). However, MDA levels were significantly decreased in the LbGp, Lip-1, and LbGp + Lip-1 groups (all *P* < 0.01) compared with the vehicle group. When Erastin was added, MDA levels were significantly increased compared with the LbGp, Lip-1, and LbGp + Lip-1 groups (all *P* < 0.01).

### *Lycium barbarum* glycopeptide and Liproxstatin-1 treatment suppress inflammation after middle cerebral artery occlusion

**Figure 9:**
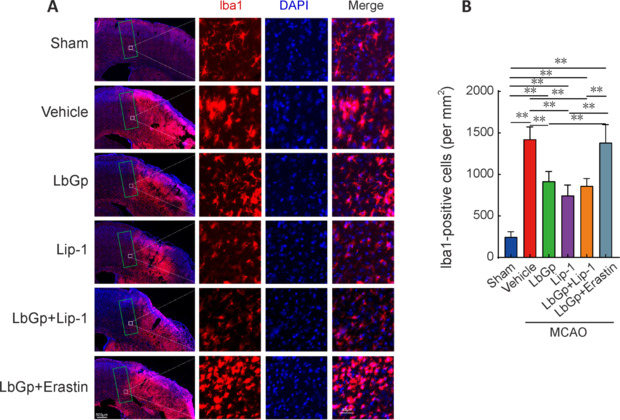
Neuroinflammatory response in the ischemic penumbra of rats in the different groups. (A) Immunofluorescence images displaying Iba1/DAPI/merge staining in the cortical penumbra. (B) Quantitation of Iba1-positive cells. The results are presented as mean ± SD (*n* = 3), **P < 0.01. DAPI: 4′,6-Diamidino-2-phenylindole; Erastin: a ferroptosis activator; Iba1: ionized calcium-binding adapter molecule 1; LbGp: *Lycium barbarum* glycopeptide; Lip-1: liproxstatin-1 (a ferroptosis inhibitor).

Immunofluorescence staining was performed in phase three to investigate inflammation in the various treatment groups after MCAO. Both the vehicle group and the LbGp + Erastin group exhibited strong Iba1 staining in the ischemic penumbra of the cortex, compared with weaker staining seen in the LbGp, Lip-1, and LbGp + Lip-1 groups (**Figure 9A**). The number of Iba1-positive cells in the vehicle group was significantly increased compared with the sham group (*P* < 0.01; **Figure 9B**). Conversely, there was a significant reduction in the number of Iba1-positive cells in the LbGp, Lip-1, and LbGp + Lip-1 groups compared with the vehicle group (*P* < 0.01). When Erastin was introduced, the number of Iba1-positive cells increased compared with the three treatment groups (*P* < 0.01). These findings collectively indicate that LbGp effectively mitigates the inflammatory response following MCAO. However, its anti-inflammatory effects are compromised when the ferroptosis inducer, Erastin, is administered alongside it.

## Discussion

The results of the present study demonstrate that treatment with LbGp reduced infarct volume and neuronal death, improved neurological function, and alleviated depression-like behaviors and memory impairment in MCAO rats. The key finding of the study is that LbGp effectively reduced the iron content in ischemic brains by downregulating the expression of TFR1 and DMT1, which are responsible for iron ion influx, and upregulating the expression of FPN1, which is responsible for iron ion efflux. Additionally, LbGp not only increased the expression of antioxidant proteins such as SLC7A11, GSH, GPX4, and SOD, but also decreased the expression of oxidant proteins and products such as ACSL4 and MDA.

### Magnetic resonance imaging provides advanced techniques to track changes in infarct size in ischemic brains

Because establishment of the MCAO rat model is not always successful, MRI imaging is used to exclude animals (no infarction or excessive infarction) from ischemic stroke studies (Gerriets et al., 2004) to ensure consistency in experimental conditions. In this study, we used MRI to analyze changes in infarct size in the early stage (24 hours after MCAO) and the late stage (30 days after MCAO) following ischemia. This approach allowed us to more precisely analyze infarct size over a month, thereby more accurately assessing the effect of LbGp on ischemic brain injury following ischemic stroke. MRI was also used to identify MCAO rats in which injury was successfully achieved. Rats with an infarct size outside of the range of 20% and 40% were excluded, which helped minimize errors associated with model variability.

### Ischemic stroke and ferroptosis

Our study demonstrated an increase in iron ion concentrations in the cortical ischemic penumbra of MCAO rats, which is consistent with many previous studies. Iron deposits have been observed in the cerebral cortex of rats subjected to ischemic stroke (Kondo et al., 1995). A significant increase in iron levels in the hippocampus has also been reported in MCAO rats (Tuo et al., 2017). Dávalos et al. (1994) reported that nearly half of patients with acute ischemic stroke exhibit elevated plasma ferritin levels, which are associated with a poor prognosis. Additionally, patients with stroke show significantly elevated plasma levels of ferritin and iron ions (Petrova et al., 2016). Iron deposition is related to destruction of the blood–brain barrier under ischemic conditions (Liu et al., 2020). Vascular rupture leads to the release of hemoglobin, transferrin, and free iron ions (Fe^3+^) from red blood cells. Fe^3+^ enters cells through TFR1 and is then reduced to Fe^2+^. DMT1 facilities entry of Fe^2+^ into the cytoplasm, while decreased FPN1 expression hinders iron efflux, leading to the accumulation of intracellular Fe^2+^ (Wei et al., 2022). Higher Fe^2+^ levels inside the cell stimulate the generation of excessive ROS via the Fenton reaction, causing lipid peroxidation and ultimately leading to cellular ferroptosis (Chen et al., 2020). In response to cellular ferroptosis, neuroinflammation occurs, which worsens brain injury (Mohan et al., 2024). Exogenous iron chelators have been shown to reduce infarct size and preserve GSH levels in ischemic rats (Demougeot et al., 2004; Millerot-Serrurot et al., 2008). In this study, we also found an increase in peroxide products, such as MDA, as well as an increase in the number of inflammatory glia, following brain ischemia. Collectively, these findings underscore iron overload as a critical factor contributing to iron-dependent cell death in ischemic brain injuries.

### Possible mechanisms by which *Lycium barbarum* glycopeptide exerts is anti-ferroptotic and antioxidant effects

In this study we found that LbGp intervenes in cellular oxidative and antioxidative processes following ischemic stroke. The antioxidative systems primarily include the System-Xc-GSH-GPX4 pathway and non-GPX4-dependent systems (Stockwell et al., 2017). To explore whether LbGp exerts its anti–iron overload and antioxidation effects via the System-Xc-GSH-GPX4 pathway in post-MCAO rats, we used a ferroptosis inhibitor, Lip-1, and a ferroptosis activator, Erastin. Lip-1 inhibits lipid peroxidation and restores GSH and GPX4 expression (Friedmann Angeli et al., 2014) and has been reported to exert protective effects in myocardial infarction and hemorrhagic stroke (Feng et al., 2019). Erastin has been widely used in cancer and other disease studies as an inducer of ferroptosis (Dixon et al., 2012; Du and Guo, 2022). Erastin functions by inhibiting System Xc, thereby diminishing intracellular cysteine levels. This, in turn, leads to a shortfall in GSH production and subsequent inactivation of GPX4, which results in an inability to adequately neutralize excessive ROS and ultimately sets the stage for ferroptosis (Sun et al., 2022).

**Figure 10:**
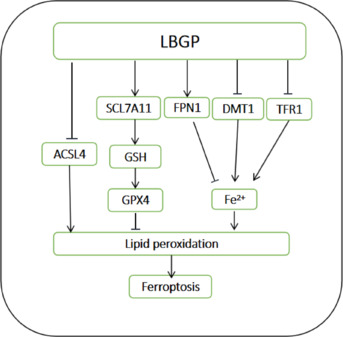
Schematic diagram illustrating the possible mechanisms by which LbGp treatment exerts its beneficial effects in middle cerebral artery occlusion rats. Arrows represent positive controls, while component symbols indicate negative controls. ACSL4: Long-chain acyl-CoA synthetase 4; DMT1: divalent metal transporter; FPN1: ferroportin 1; GPX4: glutathione peroxidase 4; GSH: glutathione; LbGp: *Lycium barbarum* glycopeptide; SCL7A11: solute carrier family 7 member 11; TFR1: transferrin receptor 1.

Our results demonstrate that LbGp resembles Lip-1 in its effects on ferroptosis and oxidation. They show similar beneficial effects in reducing ischemic brain injury and facilitating recovery of neurological function and behaviors in MCAO rats. Both LbGp and Lip-1 downregulate the expression of iron ion transporting proteins such as TFR1 and DMT1, thereby inhibiting Fe^3+^ and Fe^2+^ influx, which ultimately reduces iron ion concentrations. LbGp and Lip-1 not only upregulate the expression of anti-lipid peroxidation proteins such as SLC7A11, GSH, GPX4, and SOD but also downregulate the expression of oxidative proteins such as ACSL4 and MDA following ischemic stroke. However, these functions are partially reversed by the addition of Erastin, suggesting that LbGp and Lip-1 exert their neuroprotective effects through anti-ferroptosis pathways and anti-lipid peroxidation mechanisms, such as the System Xc-GSH-GPX4 pathway (**Figure 10**). Additionally, we showed that both LbGp and Lip-1 inhibit inflammation, which contributes to reducing ischemic brain injury.

Our results further demonstrated that LbGp is more effective than Lip-1 in improving learning and memory, as evidenced by a significant difference between the Lip-1 and the sham groups, but not between the LbGp and the sham groups on the fifth day of MWM training. Furthermore, LbGp was better able to regulate the iron ion balance in cells after stroke, due to its additional function in upregulating FPN1 expression, which facilitates the efflux of iron ions from cells. Interestingly, when both LbGp and Lip-1 were used in combination, no synergistic effect was observed, implying that they may target overlapping pathways.

Since LbGp is derived from *Lycium barbarum* polysaccharides, it retains the primary functions of LBP, such as immune modulation and inhibition of inflammation (Xu et al., 2024). LbGp has been reported to inhibit lipogenesis in glioblastoma by downregulating SREBP1c (Yao et al., 2023). Administration of LbGp can extend the lifespan and health span of *C. elegans* (Zheng et al., 2023). It also regulates autophagy to mitigate the renal and testicular injury (Zhou et al., 2022). In this study, LbGp demonstrated anti-ferroptotic, anti-lipid oxidation, and anti-inflammatory properties. Its anti-anxiety and anti-depression effects in ischemia are similar to those under in stress (Dai et al., 2023).

## Limitations

In this study, we evaluated the effect of LbGp on ischemic brain injury after MCAO and explored its underlying mechanisms. Although we only administered LbGp for 7 days compared with the 2-week administration period in a mouse model of renal and testicular injury (Zhou et al., 2022), we carried out three experimental phases to minimize variation. We selected the optimal ischemic duration and LbGp dose during phases one and two, which provided support for the feasibility of the formal experiment (phase 3). Our findings indicate that administration of LbGp for 7 consecutive days has a therapeutic effect on ischemic brain injury. However, longer treatment periods, such as 2 weeks or 1 month, and more therapeutic time points should be tested to determine the side effects of LbGp and identify the optimal therapeutic window. Furthermore, future research should include additional groups such as LbGp pre-treatment and LbGp pre-post-treatment groups, as well as dose-dependence experiments, to determine the most effective approach for treating cerebral ischemia.

## Conclusions

Our study highlights the promising therapeutic potential of LbGp as a neuroprotective agent targeting ferroptosis, oxidation, and inflammation to reduce ischemic brain damage.

## Data Availability

*No additional data are available*.
